# Avaliação do Impacto da Implantação de um Sistema de Ambulância Pré-Hospitalar sobre Mortalidade por Infarto Agudo do Miocárdio em um País em Desenvolvimento

**DOI:** 10.36660/abc.20210953

**Published:** 2022-09-15

**Authors:** Rodrigo Costa Pereira Vieira, Milena Soriano Marcolino, Luis Gustavo Silva e Silva, Daniella Nunes Pereira, Bruno Ramos Nascimento, Alzira de Oliveira Jorge, Antonio Luiz P Ribeiro

**Affiliations:** 1 Faculdade de Medicina Hospital Universitário Universidade Federal de Minas Gerais Belo Horizonte MG Brasil Faculdade de Medicina e Hospital Universitário , Universidade Federal de Minas Gerais , Belo Horizonte , MG – Brasil; 2 Rede de Telessaúde de Minas Gerais Belo Horizonte MG Brasil Rede de Telessaúde de Minas Gerais , Belo Horizonte , MG – Brasil

**Keywords:** Infarto do Miocárdio, Serviços Médicos de Emergência, Mortalidade Hospitalar

## Abstract

**Fundamento:**

O manejo efetivo de pacientes com infarto agudo do miocárdio (IAM) é tempo-dependente.

**Objetivos:**

Avaliar os impactos da implantação do atendimento pré-hospitalar nas taxas de internação e de mortalidade associadas ao IAM.

**Métodos:**

Estudo retrospectivo e ecológico, que avaliou dados do Sistema Único de Saúde, de todos os 853 municípios de Minas Gerais, de 2008 a 2016. A assimetria excessiva da mortalidade geral e intra-hospitalar por IAM foi suavizada usando o método empírico de Bayes. Este estudo avaliou a relação entre o do Serviço de Atendimento Médico de Urgência (SAMU) em cada município e os seguintes 3 desfechos: taxa de mortalidade geral por IAM, taxa de mortalidade intra-hospitalar por IAM e taxa de internação por IAM, utilizando o modelo hierárquico de Poisson. As taxas foram corrigidas pela estrutura etária e destendenciadas pela sazonalidade e influências temporais. Foi adotado um intervalo de confiança de 95%.

**Resultados:**

As taxas de mortalidade por IAM diminuíram ao longo do estudo, em média 2% por ano, com variação sazonal. A mortalidade intra-hospitalar também apresentou tendência de queda, de 13,81% em 2008 para 11,43% em 2016. A implantação do SAMU foi associada à diminuição da mortalidade por IAM ( *odds ratio* [OR] = 0,967, IC 95% 0,936 a 0,998) e mortalidade intra-hospitalar por IAM (OR = 0,914, IC 95% 0,845 a 0,986), sem associação significativa com internações (OR 1,003, IC 95% 0,927 a 1,083).

**Conclusão:**

A implantação do SAMU esteve associada a uma redução modesta, mas significativa, na mortalidade intra-hospitalar. Esse achado reforça o papel fundamental do cuidado pré-hospitalar no cuidado do IAM e a necessidade de investimentos nesse serviço para melhorar os desfechos clínicos em países de baixa e média renda.

## Introdução

O infarto agudo do miocárdio (IAM) continua sendo uma das principais causas de morbimortalidade ao redor do mundo. ^1 , 2^ O manejo efetivo dos pacientes com IAM está diretamente ligado ao tempo para assistência médica e aproximadamente metade dos óbitos atribuídos a IAM resultam de parada cardíaca fora do hospital, reforçando a importância do atendimento pré-hospitalar e do desenvolvimento de sistemas de atenção para IAM baseados em evidências.

Especificamente, em relação ao infarto do miocárdio com supradesnivelamento do segmento ST, o diagnóstico precoce e a terapia de reperfusão adequada são de extrema importância para a redução da mortalidade. A intervenção coronária percutânea (ICP) primária, primeira escolha para reperfusão, quando disponível, é mais eficaz do que a terapia trombolítica, mas idealmente deve ser realizada até 120 minutos após o primeiro contato médico, ou em até 90 minutos se o paciente for atendido em uma unidade capaz de PCI primária. A trombólise é mais eficaz quando administrada dentro de 3 horas do início dos sintomas. Qualquer uma das estratégias deve ser administrada em até 12 horas após o início dos sintomas. ^3^ Na prática clínica, uma proporção significativa de pacientes não recebe atendimento que cumpra essas metas de tempo e a situação é ainda pior em comunidades rurais e áreas com poucos recursos. ^3 - 7^

Ainda faltam dados contemporâneos sobre o impacto do uso do atendimento pré-hospitalar no cenário do IAM, especialmente em países de baixa e média renda, principalmente na perspectiva mais generalizável de uma investigação de base comunitária, bem como informações sobre os resultados hospitalares de pacientes transportados por ambulância. No entanto, sabe-se que os primeiros minutos após o início do IAM são cruciais para o prognóstico e sobrevida do paciente e, considerando isso, é importante avaliar objetivamente os possíveis impactos do atendimento pré-hospitalar de emergência no manejo e nos desfechos do IAM. ^8^ Porém, é difícil obter esse tipo de informação a partir de estudos observacionais, pois não há controle sobre a classificação das variáveis estudadas e, muitas vezes, é difícil isolar a variável dependente.

Portanto, objetivamos avaliar os impactos da implantação do Serviço de Atendimento Médico de Urgência (SAMU) nas taxas de internação e na mortalidade geral e hospitalar por IAM no estado de Minas Gerais (MG), na Região Sudeste do Brasil.

## Métodos

Trata-se de um estudo observacional, retrospectivo e ecológico, que avaliou dados do Sistema Único de Saúde (SUS, DataSUS TabNET), ^9^ de todos os 853 municípios do estado de MG, no período de 2008 a 2016, de acordo com a declaração RECORD para estudos que usam dados de saúde coletados rotineiramente. ^10^

MG é o estado com maior número de municípios (853) no Brasil, estando localizado na Região Sudeste do Brasil. É o segundo estado mais populoso do Brasil, com 21 milhões de habitantes distribuídos em uma área comparável à da França. O Índice de Desenvolvimento Humano (IDH) médio é de 0,731 e 14,46% da população é considerada pobre ou muito pobre, segundo o Instituto Brasileiro de Geografia e Estatística (IBGE). ^11^ MG pode ser considerada representativa do país, pois a distribuição etária, o percentual de urbanização e a desigualdade social são semelhantes ao padrão nacional geral. As regiões norte e nordeste de MG apresentam os menores IDH, semelhantes às regiões Norte e Nordeste do Brasil, enquanto as regiões oeste e sul do estado apresentam IDH semelhante às áreas de maior IDH do país. ^10 - 12^

**Figura 1:**
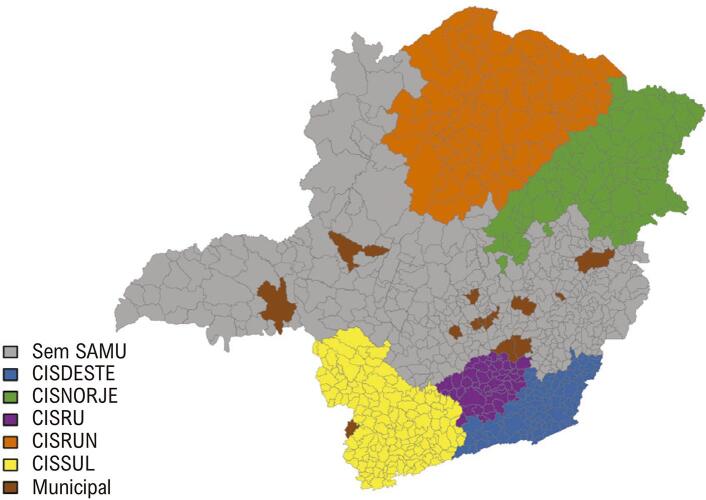
Distribuição de SAMUs municipais e consórcios de SAMU em Minas Gerais. Acrônimos: CISDESTE: Consórcio Intermunicipal de Saúde da Região Sudeste; CISNORJE: Consórcio Intermunicipal de Saúde Rede de Urgência Macro Nordeste/Jequitinhonha; CISRU: Consórcio Intermunicipal de Saúde da Rede de Urgência Centro-Sul; CISRUN: Consórcio Intermunicipal de Saúde da Rede de Urgência do Norte de Minas; CISSUL: Consórcio Intermunicipal de Saúde da Macrorregião do Sul de Minas; SAMU: Serviço de Atendimento Médico de Urgência. Fonte: SES-MH 2016.

O serviço nacional de ambulâncias, denominado SAMU, foi implantado no estado de MG em 2003 por um programa nacional denominado “Política Nacional das Urgências”. O programa teve início em alguns municípios selecionados, que ficaram responsáveis pela gestão de seu próprio sistema. A partir de 2009 foram criados sistemas de atendimento pré-hospitalar regionalizado, denominados “consórcios”, que atualmente correspondem ao modelo principal de atendimento e abrangem diversas regiões do estado. No período analisado, havia 5 consórcios no estado: Consórcio Intermunicipal de Saúde da Macrorregião do Sul de Minas (CISSUL), Consórcio Intermunicipal de Saúde da Região Sudeste (CISDESTE), Consórcio Intermunicipal de Saúde da Rede de Urgência Centro Sul (CISRU), Urgência do Norte de Minas (CISRUN) e Consórcio Intermunicipal de Saúde Rede de Urgência Macro Nordeste/Jequitinhonha (CISNORJE), abrangendo 469 municípios nas regiões sul, sudeste, centro-sul, norte e nordeste, respectivamente ( Figura 1 ).

As datas de implantação do SAMU foram obtidas junto ao governo estadual e coordenadores locais do SAMU. O primeiro consórcio intermunicipal foi implantado em 2009.Em 2011, foi implantado um, em 2012, dois e em 2015, mais um. Alguns serviços foram implantados antes do período analisado, enquanto outros foram implantados no período do estudo. Ao mesmo tempo, alguns municípios já haviam implantado consórcios de SAMU no início da análise, enquanto outros implantaram o SAMU durante o período do estudo e outros não possuíam SAMU durante esse período. Catorze municípios possuíam SAMU no período analisado (SAMU municipais). O SAMU foi implantado em 86 municípios em janeiro de 2009 (CISRUN), em 94 municípios em novembro de 2014 (CISDESTE), em 86 municípios em abril de 2012 (CISNORJE), em 51 municípios em junho de 2012 (CISRU) e em 152 municípios em julho de 2015 (CISSUL). Os demais municípios do estado (n = 370) permaneceram sem SAMU durante todo o período do estudo. A Figura Suplementar 1 mostra a evolução da cobertura do SAMU no estado, à medida que cada consórcio regional foi implementado.

Os desfechos de interesse foram as taxas de mortalidade geral e intra-hospitalar por IAM e taxa de internações por IAM, avaliadas no período de 2008 a 2016. A escolha desses desfechos justifica-se pelo fato de serem de maior relevância clínica e epidemiológica e de maior potencial de associação com a implantação do SAMU, além das altas taxas de preenchimento, enquanto variáveis obrigatórias.

Os dados sobre a população de cada município foram obtidos do IBGE, o Instituto Demográfico e Estatístico oficial do Brasil. ^13^ Para óbitos e internações, os dados foram extraídos do DATASUS TabNET, ^9^ um banco de dados eletrônico que coleta informações em nível de paciente do SUS. Foram utilizadas informações de ocorrências mensais desses desfechos, das bases de dados do Sistema de Informações sobre Mortalidade (SIM) e do Sistema de Informações Hospitalares (SIH), respectivamente, para a população dos 853 municípios de MG, de 2008 a 2016. ^14^

Os óbitos foram considerados óbitos por IAM quando a causa principal do óbito apresentava os seguintes códigos da CID-10 (I21 a I24): I21 “infarto agudo do miocárdio”, I22 “infarto do miocárdio recorrente”, I23 “algumas complicações atuais subsequentes a infarto agudo do miocárdio”, I24 “outras cardiopatias isquêmicas agudas”. Os dados do SIH foram utilizados para obtenção dos dados das internações por IAM: procedimento “tratamento do IAM”, código SIH/DATASUS 03.03.06.019-0 e “angioplastia coronária primária”, código 040.603.004-9. Neste estudo, a mortalidade intra-hospitalar por IAM foi calculada a partir do número de óbitos pelos códigos citados, dividido pelo número de internações por esses mesmos códigos em cada município por mês de análise.

Como o estudo utilizou dados públicos disponíveis na plataforma DATASUS, não foi necessária a aprovação por um comitê de ética em pesquisa.

### Análise de dados

Foi utilizado o software R versão 3.3.4 para análise estatística. ^15^ A unidade de análise foi o município. Foi realizada uma análise mensal dos desfechos, de janeiro de 2008 a dezembro de 2016, para cada um dos 853 municípios de MG, considerando a população por estimativas de cada ano. ^15^

Os 3 desfechos foram ajustados pela estrutura etária, com base na população de 2010. ^11^ As taxas foram estimadas para cada município e mês considerando o ajuste por idade. Os municípios foram indexados por i = 1, …, n, onde n = 853 é o número total de municípios, enquanto os meses foram indexados por t = 1, …, T, onde T é o número de meses no período analisado.

A assimetria excessiva da mortalidade geral e intra-hospitalar por IAM foi suavizada pelo método empírico de Bayes. ^16^ O método foi usado para estimar as taxas em vez da abordagem clássica de estimativa de taxas. Na abordagem clássica, a taxa é calculada como a razão do número de eventos (óbitos, internações) pela população sob risco. Portanto, a variabilidade das taxas estimadas é fortemente afetada por pequenas mudanças no número de eventos (óbitos) quando computados para pequenas áreas onde o valor esperado para eventos é baixo. O método empírico de Bayes visa minimizar as variações das taxas estimadas por meio de uma média ponderada entre a taxa municipal e a taxa regional. No presente estudo, a região foi definida como o estado de MG. Os pesos foram interpretados pelo tamanho da população; quanto maior a população, menor o peso da taxa de região. As taxas de mortalidade e hospitalização por IAM foram modeladas usando a distribuição de Poisson, enquanto a mortalidade intra-hospitalar por IAM foi pela distribuição binomial. Em seguida, foram estimadas pelo método empírico de Bayes (Figuras Suplementares 2 e 3). Foram avaliadas as internações com os referidos códigos de internação do SIH/DATASUS de 2008 a 2016 em todos os municípios de MG e, como essa variável também está sujeita a variações significativas em municípios com pequenas populações, foi utilizado para ajuste o método empírico global de Bayes, conforme explicado para análise da taxa de mortalidade.

Foram observadas tendências sazonais e temporais de queda das taxas de mortalidade geral e hospitalar, com tendência semianual na oscilação entre as taxas mais baixas e mais altas e redução gradativa das mesmas ao longo do período analisado. Portanto, a sazonalidade e a temporalidade foram incluídas nos modelos de análise estatística. O presente estudo avaliou a relação entre a disponibilidade de atendimento do SAMU em cada município e os seguintes 3 desfechos: mortalidade por IAM na população geral, mortalidade por IAM intra-hospitalar e número de internações por IAM, utilizando o modelo hierárquico de Poisson, e as taxas analisadas foram corrigidas pela estrutura etária e destendenciadas por influências temporais e sazonais. Um intervalo de confiança de 95% (IC) foi usado para todos os resultados.

## Resultados

**Figura 2:**
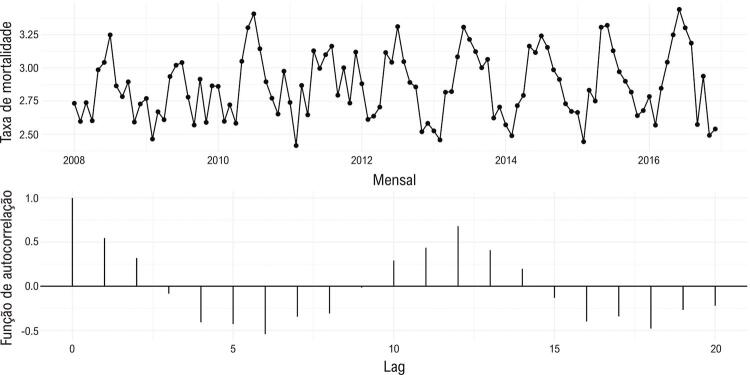
Ocorrência de variação sazonal nas taxas de mortalidade de 2008 a 2016. Uma maior taxa de mortalidade é observada durante o inverno e taxas mais baixas são observadas durante o verão.

As taxas de mortalidade por IAM, ajustadas pela distribuição etária, apresentaram tendência decrescente ao longo do estudo, variando de 35,7 óbitos por 100.000 habitantes em 2008 a 30,4 óbitos por 100.000 habitantes em 2016, ou seja, cerca de 2% por ano em média ( Tabela 1 ). Também foi observada variação sazonal nas taxas de mortalidade por IAM, sendo maior no inverno e menor no verão ( Figura 2 ).

**Tabela 1:** Taxas anuais de mortalidade, internação e mortalidade intra-hospitalar por IAM, ajustadas por idade, no estado de Minas Gerais, de 2008 a 2016

Ano	Mortalidade (por 100.000)	Mortalidade intra-hospitalar (%)*	Internações (por 100.000)
2008	35,7 (35,3 – 36,1)	13,81	152 (146 - 158)
2009	34,1 (33,8 – 34,5)	13,65	150 (144 - 156)
2010	35,0 (34,6 – 35,3)	13,78	140 (134 - 145)
2011	33,8 (33,4 – 34,1)	11,82	147 (142 - 152)
2012	32,4 (32,1 – 32,6)	11,29	146 (141 - 151)
2013	31,9 (31,7 – 32,2)	11,99	142 (137 - 146)
2014	30,9 (30,6 – 31,1)	12,15	137 (132 - 141)
2015	29,9 (29,7 – 30,2)	10,82	138 (133 - 142)
2016	30,4 (30,1 – 30,6)	11,43	147 (142 - 151)

*As taxas são expressas com intervalo de confiança de 95%.

**Figura 3:**
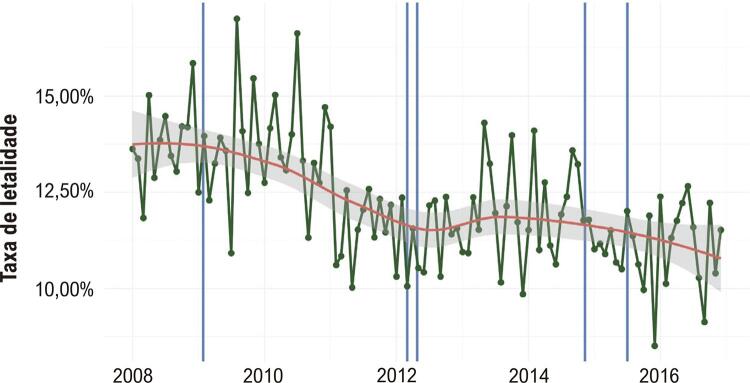
Taxas de letalidade e mortalidade mensais para os municípios de Minas Gerais, de 2008 a 2016. Os pontos verdes representam taxas mensais de letalidade para toda a população analisada, que apresenta variação mensal considerável sem padrão sazonal definido. A linha vermelha representa a tendência da taxa ao longo do tempo, com uma redução acentuada de 2008 para 2012, estagnação em 2013 e uma nova redução mais lenta de 2014 para 2016. Observamos que a implantação do Serviço de Atendimento Médico de Urgência (SAMU), representado pelas linhas verticais azuis, não teve influência clara na letalidade na análise descritiva.

A mortalidade intra-hospitalar por IAM corrigida pela idade também apresentou tendência decrescente, de 13,81% em 2008 para 11,43% em 2016 ( Tabela 1 ), com amplas variações mensais e padrão sazonal, embora menos evidente quando comparada à taxa de mortalidade por IAM ( Figura 3 ).

A implantação do SAMU foi associada à diminuição da mortalidade por IAM ( *odds ratio* [OR] = 0,967, IC 95% 0,936 a 0,998) e mortalidade intra-hospitalar por IAM (OR = 0,914, IC 95% 0,845 a 0,986), sem associação significativa com o número de internações (OR 1,003, IC 95% 0,927 a 1,083). Não houve variação sazonal no número de internações por IAM durante o período do estudo.

## Discussão

No presente estudo, foi observado que a implantação do SAMU no estado brasileiro de MG esteve associada à redução das taxas gerais de mortalidade por IAM. A mortalidade intra-hospitalar por IAM corrigida pela idade também apresentou tendência decrescente ao longo do tempo, com padrão sazonal. No entanto, não foi encontrada associação significativa com as taxas de internação por IAM no período analisado.

Apesar da falta de evidências em países em desenvolvimento, esses resultados estão de acordo com estudos desenvolvidos em países de alta renda, e provavelmente estão relacionados ao maior acesso do paciente a algum tipo de tratamento do IAM e à diminuição do tempo entre o início dos sintomas e o início da terapia específica, incluindo reperfusão quando indicada. ^3 , 8 , 17 , 18^ Por outro lado, alguns estudos não conseguiram demonstrar associação entre a implantação de sistemas de atenção ao IAM e diminuição da mortalidade, apesar da melhora na qualidade da assistência, adesão às recomendações guiadas por diretrizes e tempo de tratamento reduzidos. ^3 - 5 , 8 , 12 , 19 , 20^ Isso ocorreu mesmo com o uso de protocolos elaborados com múltiplas intervenções além do atendimento pré-hospitalar. Dentre esses estudos, destaca-se o Reperfusion of Acute myocardial infarction in North Carolina Emergency departments (RACE). Foi realizado no estado da Carolina do Norte, EUA, levando à redução do tempo de consulta e dos atrasos na administração do tratamento de reperfusão após a implantação e sistematização de protocolos de atendimento ao paciente com IAM. ^20^

Diferentemente de relatos anteriores, nosso estudo foi realizado em um país de renda média, fato que pode ajudar a explicar nossos resultados positivos em termos de desfechos duros. Em estudos realizados em países desenvolvidos, a qualidade de linha de base do atendimento ao IAM é geralmente superior à observada em nosso país. ^6 , 8 , 20^ Assim, demonstrar a magnitude do benefício incremental de qualquer intervenção é mais difícil nessa situação, enquanto em locais onde há baixa qualidade e prestação de cuidados, intervenções menos complexas podem ter um impacto maior nos resultados. Uma avaliação prévia da adesão aos critérios de qualidade da atenção ao IAM em Minas Gerais ilustra essa lacuna assistencial na região. ^21 - 23^ Apesar da implantação do atendimento pré-hospitalar não fazer parte da implementação dos sistemas de atenção ao IAM, as unidades intervencionistas ou unidades especializadas em cardiologia nessas regiões, pode ter influenciado na melhora da qualidade do atendimento no período do estudo, uma vez que maior proporção de pacientes pode ter recebido diagnóstico preciso e tratamento adequado.

Os desfechos associados ao IAM estão relacionados a múltiplas variáveis, principalmente a estrutura para o atendimento dos pacientes. ^22 - 24^ Vale destacar alguns elementos relevantes na formulação de uma rede de alta complexidade em cardiologia: 1) a relação inversa entre o tamanho e volume de procedimentos realizados em hospitais de referência e mortalidade por IAM; 2) benefício da estratégia “farmaco-invasiva”, que consiste em fibrinólise pré-hospitalar ou intra-hospitalar precoce seguida de ICP de rotina entre 3 e 24 horas, comparável à ICP primária em pacientes com sintomas de IAM de curta duração, sempre que for possível a transferência oportuna para unidades com capacidade de ICP; ^19^ 3) custo-efetividade da expansão do atendimento pré-hospitalar em relação à construção de novos serviços de cardiologia intervencionista, que foi demonstrada nos EUA, onde a distância até os serviços de hemodinâmica é em torno de 70 km na grande maioria dos locais. ^1 , 2 , 20^

**Figura 4:**
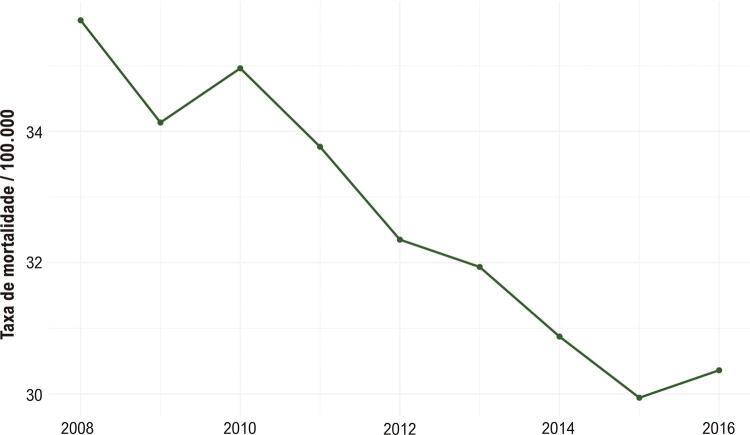
Taxas anuais de mortalidade bruta no estado de Minas Gerais de 2008 a 2016. Uma tendência de redução temporal.

Como esperado, houve maior variação de taxas em locais com menor população, como nas regiões leste e centro-sul do estado, que possuem os menores IDH do estado, respectivamente cobertos pelos consórcios CISDESTE e CISRU. Esse achado possivelmente está associado a menor qualidade do preenchimento da declaração de óbito, com maior proporção de óbitos por causas indefinidas e “códigos lixo” (condições de saúde que não podem ser diretamente atribuíveis à mortalidade), associados à influência de outras causas de mortalidade nessas populações, como as doenças infecciosas, condizente com o observado em outras regiões de baixo IDH no Brasil e mundialmente (OMS, 2017). ^24 - 26^ Esse achado reitera a importância do uso de métodos de ajuste analítico para variações extremas. Nesse sentido, a análise metodológica com a aplicação do método de Bayes empírico global foi eficaz em reduzir a variação das taxas em pequenas populações, sem alterar os valores em locais com maiores populações, conforme mostra a Figura 4 . A utilização das taxas observadas no estado de MG como referência para suavização permitiu que esse processo fosse feito com uma população de referência semelhante aos municípios analisados, mas com uma população maior.

Com relação à variação sazonal das taxas de mortalidade por IAM, essa variação não era esperada em uma região com variação de temperatura muito menor em comparação com países da América do Norte ou da Europa, ou mesmo com regiões mais frias do Brasil, como São Paulo e a Região Sul. Levanta a possibilidade de outras explicações além da temperatura, ou alterações fisiológicas relacionadas à temperatura, como alterações na viscosidade do sangue, volume plaquetário e pressão arterial. ^27^ Outras doenças mais comuns durante o inverno, especialmente infecções respiratórias por vírus e pneumonia adquirida na comunidade demonstrou afetar o sistema cardiovascular de várias maneiras e precipitar desfechos cardíacos adversos, como insuficiência cardíaca, infarto do miocárdio e arritmias cardíacas. ^28^ Como a doença arterial coronariana é essencialmente uma doença inflamatória, foi demonstrado que a inflamação relacionada a patógenos respiratórios pode desencadeá-la. ^29^ Em uma série de casos autocontrolados usando dados nacionais de vigilância de infecções, vinculada ao Scottish Morbidity Record, as taxas de IAM e acidente vascular cerebral aumentaram substancialmente na semana seguinte a uma infecção respiratória. ^30^ Além disso, uma meta-análise recente mostrou o impacto da vacina pneumocócica polissacarídica 23-valente em fornecer proteção contra qualquer evento cardiovascular ( *risk ratio* [RR]: 0,91; IC 95%: 0,84 a 0,99), infarto do miocárdio (RR: 0,88; IC 95%: 0,79 a 0,98) e mortalidade por todas as causas (RR: 0,78; IC 95%: 0,68 a 0,88) em indivíduos de todas as faixas etárias. ^31^ Por outro lado, um ensaio clínico em andamento realizado no Brasil comparando vacina contra influenza em dose única e em dose dupla após um evento coronariano agudo foi interrompido precocemente devido à aparente falta de benefício nas análises provisórias. ^32^

A poluição do ar, especialmente a exposição ao material particulado, também tem sido associada a um maior risco de doenças cardiovasculares, incluindo IAM. ^33 , 34^ Um estudo recente observou que um aumento de cerca de 10 microgramas por m ^3^ de ar foi associado a um aumento de 16% de mortalidade por IAM. ^33^ Enquanto isso, outro estudo usando um modelo global de química atmosférica mostrou que mais de 60% das mortes cardiovasculares ao redor do mundo estão relacionadas à poluição do ar, e aumentos de curto prazo na poluição do ar estão associados ao IAM. ^34^ Sunyer et al., observaram que o aumento dos níveis de dióxido de enxofre no ar de sete cidades europeias aumentou as internações por doenças cardiovasculares no dia anterior e no dia de maior teor de poluentes. ^35^ Essa associação permaneceu significativa mesmo após o ajuste para partículas com tamanho inferior a 10 μm entre indivíduos com menos de 65 anos. A poluição do ar tende a ser maior nos meses em que os níveis de chuva são mais baixos. Portanto, no Brasil, onde os verões são chuvosos e os invernos predominantemente mais secos, a sazonalidade pode, de fato, ter um papel importante na mortalidade por IAM.

Nosso estudo tem algumas limitações que devem ser mencionadas. A implementação do atendimento pré-hospitalar é acompanhada de co-intervenções, como a articulação da vinculação das instituições de saúde do sistema de atenção ao IAM e aumento dos serviços locais de emergência, que não foram sistematicamente estudados nesta pesquisa e podem ter influenciado os achados. Por meio de informações públicas disponibilizadas pelo Ministério da Saúde, Secretaria de Estado de Saúde de Minas Gerais, Secretarias Municipais de Saúde e bancos de dados dos SAMUs, é possível afirmar que as redes disponíveis eram incipientes e que havia falta de infraestrutura, insumos, recursos humanos e processos organizacionais e de administração na maior parte do estado e do país. ^11 , 13 , 14^ Exemplos incluem a baixa disponibilidade de serviços de cardiologia intervencionista e a concentração de recursos de saúde em regiões com melhores perfis sociodemográficos; a baixa utilização dos serviços de telessaúde pelas equipes pré-hospitalares; a ausência de fibrinolíticos nas ambulâncias do SAMU; a falta de protocolos clínicos e padronização de cuidados baseados em evidências; a infraestrutura precária da maioria dos departamentos de emergência do estado, principalmente unidades de emergência pré-hospitalar e hospitais de pequeno porte; e o treinamento insuficiente do pessoal de saúde envolvido no atendimento de emergência. Por fim, esses fatores somam-se à dificuldade de implantação das redes de atenção à saúde por motivos financeiros e políticos.

O presente estudo não abordou o impacto de outros níveis de atenção nos desfechos estudados, como a atenção primária à saúde (APS). Embora existam dados consistentes na literatura mostrando o papel fundamental da APS na prevenção, promoção e tratamento de condições de saúde que são fatores de risco para IAM, com sua capacidade de reduzir as taxas de incidência de IAM, o foco deste estudo foram eventos agudos que ocorrem, embora em proporção menor, em sistemas de APS bem estabelecidos. ^1 , 2 , 6 , 8 , 20^ Como os efeitos na saúde associados à APS são tipicamente observados a longo prazo, não foi feito ajuste para essa variável, dadas as dificuldades técnicas inerentes a esse processo e a escassez de bases de dados para realizá-lo.

Outra limitação está relacionada ao fato de o modelo ecológico não incluir variáveis clínicas individuais relevantes; portanto, não é possível estabelecer relações entre tais características e os desfechos estudados ou estabelecer uma relação causal definitiva entre a implantação do SAMU e esses desfechos. Finalmente, como todos os estudos observacionais, o risco de viés foi minimizado, mas não pode ser completamente excluído, em particular o viés de confusão residual.

No entanto, o maior ponto forte do nosso estudo é sua contribuição metodológica, ou seja, apresenta um método capaz de levar em conta a sazonalidade e as tendências temporais para observar o efeito de uma intervenção. A tendência temporal de redução da mortalidade foi considerada na análise e foi observada a redução da mortalidade com a implantação do SAMU independentemente dessa tendência temporal.

## Conclusão

No presente estudo, observou-se uma pequena redução nas taxas de mortalidade geral e hospitalar atribuíveis ao IAM após a implantação do SAMU em MG no período analisado, sem alterações significativas nas taxas de internação hospitalar. Os resultados sugerem que o atendimento pré-hospitalar desempenha um papel importante no sistema de saúde, especialmente considerando a crescente carga de doenças cardiovasculares, especialmente síndromes coronarianas agudas.

## * Material suplementar

Para informação adicional, por favor, clique aqui.
