# Supplementary material for: Avaliação do Impacto da Implantação de um Sistema de Ambulância Pré-Hospitalar sobre Mortalidade por Infarto Agudo do Miocárdio em um País em Desenvolvimento
Source: Arq Bras Cardiol. 2022 Sep 15;119(5):756–63. [Article in Portuguese] doi: 10.36660/abc.20210953 (PMC9750209; doi:10.36660/abc.20210953)
Supplement: Translation

# Translation: Assessment of the Impact of the Implementation of a Pre-Hospital Ambulance System on Acute Myocardial Infarction Mortality in a Developing Country

**Authors:** Rodrigo Costa Pereira Vieira, Milena Soriano Marcolino, Luis Gustavo Silva e Silva, Daniella Nunes Pereira, Bruno Ramos Nascimento, Alzira de Oliveira Jorge, Antonio Luiz P Ribeiro

**Affiliations:** 1 Brazil Faculdade de Medicina e Hospital Universitário, Universidade Federal de Minas Gerais, Belo Horizonte, MG – Brazil; 2 Brazil Rede de Telessaúde de Minas Gerais, Belo Horizonte, MG – Brazil

**Keywords:** Acute Myocardial Infarction, Emergency Medical Services, Hospital Mortality

## Abstract

**Background:**

The effective management of patients with acute myocardial infarction (AMI) is time-dependent.

**Objectives:**

To assess the impacts of the implementation of prehospital care on admission rates and mortality associated with AMI.

**Methods:**

Retrospective, ecological study, which assessed data from the Brazilian Universal Health System, from all 853 municipalities of Minas Gerais, from 2008 to 2016. Excessive skewness of general and in-hospital mortality rates was smoothed using the empirical Bayes method. This study assessed the relationship between Mobile Emergency Care Service (SAMU) in each municipality and the following 3 outcomes: mortality rate due to AMI, AMI in-hospital mortality, and AMI hospitalization rate, using the Poisson hierarchical model. Rates were corrected by age structure and detrended by seasonality and temporal influences. A confidence interval of 95% was adopted.

**Results:**

AMI mortality rates decreased throughout the study, on average 2% per year, with seasonal variation. AMI in-hospital mortality also showed a decreasing trend, from 13.81% in 2008 to 11.43% in 2016. SAMU implementation was associated with decreased AMI mortality (odds ratio [OR] = 0.967, 95% confidence interval [CI] 0.936 to 0.998) and AMI in-hospital mortality (OR = 0.914, 95% CI 0.845 to 0.986), with no relation with hospitalizations (OR = 1.003, 95% CI 0.927 to 1.083).

**Conclusion:**

SAMU implementation was associated with a modest but significant decrease in AMI in-hospital mortality. This finding reinforces the key role of prehospital care in AMI care and the need for investments on this service to improve clinical outcomes in low- and middle-income countries.

## Introduction

Acute myocardial infarction (AMI) remains a leading cause of morbidity and mortality worldwide.^1 , 2^ The effective management of patients with AMI is directly linked to time to medical assistance, and approximately one half of the deaths attributed to AMI result from out-of-hospital cardiac arrest, reinforcing the importance of prehospital care and, ultimately, the development of evidence-based AMI systems of care.

Specifically, in relation to ST-elevation myocardial infarction, early diagnosis and appropriate reperfusion therapy are of the utmost importance for mortality reduction. Primary percutaneous coronary intervention (PCI), the first choice for reperfusion, when available, is more effective than thrombolytic therapy, but it should ideally be performed up to 120 minutes after the first medical contact, or within 90 minutes if the patient presents at a primary PCI-capable unit. Thrombolysis is more effective when delivered within 3 hours of symptom onset. Either strategy should be delivered no later than 12 hours after symptom onset.^3^ In clinical practice, a significant proportion of patients do not receive care that meets those time targets, and the situation is even worse in rural communities and under-resourced areas.^3 - 7^

There is still a lack of contemporary data about the impact of the use of prehospital care in the setting of AMI, especially in low- and middle-income countries, particularly from the more generalizable perspective of a community-based investigation, as well as information about hospital outcomes of patients transported by ambulance. However, it is known that the first minutes after AMI onset are crucial to the patient’s prognosis and survival, and, considering that, it is important to objectively evaluate the possible impacts of emergency prehospital services on AMI management and outcomes.^8^ Nonetheless, it is difficult to obtain this type of information from observational studies, as there is no control over the classification of the studied variables, and it is often difficult to isolate the dependent variable.

Therefore, we aimed to assess the impacts of the implementation of a nationwide ambulance service ( *Serviço de Atendimento Médico de Urgência* , SAMU) on hospitalization rates and general and AMI in-hospital mortality in the state of Minas Gerais (MG), in the Southeast Region of Brazil.

## Methods

This is an observational, retrospective, ecological study, which assessed data from the Brazilian Universal Health System (SUS, DataSUS TabNET),^9^ from all 853 municipalities of the Brazilian state of MG, from 2008 to 2016. It follows the RECORD statement for studies that use routinely collected health data.^10^

MG is the Brazilian state with the largest number of municipalities (853), and it is located in the Southeast Region of Brazil. It is the second most populous state in Brazil, with 21 million inhabitants distributed across an area comparable to France. The average Human Development Index (HDI) is 0.731, and 14.46% of the population is considered poor or very poor, according to the Brazilian Institute of Geography and Statistics (IBGE).^11^ MG can be considered representative of the country, as age distribution, percentage of urbanization, and social inequality are similar to the overall national pattern. The north and the northeast of MG have the lowest HDI, similar to the North and Northeast regions of Brazil, while the west and south of the state have HDI similar to the areas with the highest HDI in the country.^10 - 12^

The Brazilian nationwide ambulance service, called SAMU, was implemented in the state of MG in 2003 by a national program called “ *Política Nacional das Urgências* ” (National Emergency Policy). The program started in some selected municipalities, which were responsible for managing their own system. Since 2009 regionalized prehospital care systems were created, called “consortiums”, which currently correspond to the main model of care and cover various regions of the state. In the period analyzed, there were five consortia in the state: Consórcio Intermunicipal de Saúde da Macrorregião do Sul de Minas (CISSUL), Consórcio Intermunicipal de Saúde da Região Sudeste (CISDESTE), Consórcio Intermunicipal de Saúde da Rede de Urgência Centro Sul (CISRU), Urgência do Norte de Minas (CISRUN) and Consórcio Intermunicipal de Saúde Rede de Urgência Macro Nordeste/ Jequitinhonha (CISNORJE), covering 469 municipalities in the south, southeast, center-south, north, and northeast regions, respectively ( Figure 1 ).

**Figure 1:** Distribution of municipal SAMUs and SAMU consortia in Minas Gerais. Acronyms: CISDESTE: Consórcio Intermunicipal de Saúde da Região Sudeste; CISNORJE: Consórcio Intermunicipal de Saúde Rede de Urgência Macro Nordeste/Jequitinhonha; CISRU: Consórcio Intermunicipal de Saúde da Rede de Urgência Centro-Sul; CISRUN: Consórcio Intermunicipal de Saúde da Rede de Urgência do Norte de Minas; CISSUL: Consórcio Intermunicipal de Saúde da Macrorregião do Sul de Minas; SAMU: Mobile Emergency Care Service. Source: SES-MH 2016.

SAMU implementation dates were obtained from the state government and local SAMU coordinators. The first inter-city consortium was implemented in 2009, one in 2011, two in 2012, and one in 2015. Some services were implemented before the analyzed time period, while others were implemented during the study period. At the same time, some municipalities had already implemented SAMU consortiums at the beginning of the analysis, while others implemented SAMU during the study period and others did not have SAMU within this time frame. Fourteen municipalities had SAMU during the analysis period (municipal SAMUs). SAMU was implemented in 86 municipalities in January 2009 (CISRUN), in 94 municipalities in November 2014 (CISDESTE), in 86 municipalities in April 2012 (CISNORJE), in 51 municipalities in June 2012 (CISRU), and in 152 municipalities in July 2015 (CISSUL). The remaining municipalities of the state (n = 370) remained without SAMU throughout the study period. Supplementary Figure 1 shows the evolution of SAMU coverage in the state, as each regional consortium was implemented.

The outcomes of interest were AMI general and in-hospital mortality rates and rate of admissions due to AMI, assessed from 2008 to 2016. The choice of those outcomes is justified by the fact that they have the greatest clinical and epidemiological relevance and greatest potential for association with SAMU implementation, in addition to high completion rates, as mandatory variables.

Data on the population of each municipality was obtained from IBGE, the official demographic and statistical institute of Brazil.^13^ For deaths and hospitalizations, data were extracted from DATASUS TabNET,^9^ an electronic database that collects patient-level information from the SUS. We used information of monthly occurrences of those outcomes, from the Mortality Information System (SIM) and the Hospital Information System (SIH) databases, respectively, for the population of the 853 municipalities of MG, from 2008 to 2016.^14^

Deaths were considered AMI deaths when the main cause of death had the following ICD-10 codes (I21 to I24): I21 “acute myocardial infarction”, I22 “recurrent myocardial infarction”, I23 “some current complications subsequent to acute myocardial infarction”, I24 “other acute ischemic heart diseases”. Data from the SIH was used to obtain data on hospital admissions due to AMI: procedure “treatment of AMI”, SIH/DATASUS code 03.03.06.019-0 and “primary coronary angioplasty”, code 040.603.004-9. In this study, AMI in-hospital mortality was calculated from the number of deaths with the aforementioned codes, divided by the number of hospitalizations with these same codes in each municipality per month of analysis.

As the study used public data available through the DATASUS platform, approval by a research ethics committee was not necessary.

### Data analysis

The software R version 3.3.4 was used for statistical analysis.^15^ The unit of analysis was the municipality. A monthly analysis of the outcomes, from January 2008 to December 2016, for each of the 853 municipalities in MG was performed, considering the population by estimates of each year.^15^

The 3 outcomes were adjusted for the age structure, based on the population of 2010.^11^ The rates were estimated for each municipality and month considering the adjustment for age. The municipalities were indexed by i = 1, …, n, where n = 853 is the total number of municipalities, while the months were indexed by t = 1, …, T, where T is the number of months in the period analyzed.

Excessive skewness of AMI mortality and in-hospital mortality rates were smoothed using the empirical Bayes method.^16^ The method was used to estimate the rates instead of the classic rate estimate approach. In the classical approach, the rate is calculated as the ratio of the number of events (deaths, hospitalizations) per the population at risk. Therefore, the variability of the estimated rates is strongly affected by small changes in the number of events (deaths) when they are computed for small areas where the expected value for events is low. The empirical Bayes method aims to minimize the variations of the estimated rates through a weighted average between the municipal rate and the regional rate. In the present study, the region was defined as the state of MG. The weights were interpreted by the population size; the higher a population was, the lesser the weight of the region rate. The AMI mortality and hospitalization rates were modeled using Poisson distribution, while AMI in-hospital mortality was using binomial distribution, and then estimated them through the empirical Bayes method (Supplementary Figures 2 and 3). Hospital admissions with the aforementioned SIH/DATASUS hospital admission codes from 2008 to 2016 in all municipalities of MG were assessed and, as this variable is also subject to significant variations in municipalities with small populations, the global empirical Bayes method was used for adjustment, as explained for mortality rate analysis.

Seasonality and temporal trends of decrease in the general and hospital mortality rates were observed, with semiannual tendency in the oscillation between the lowest and highest rates and gradual reduction of the same over the analyzed period. Thus, seasonality and temporality were included in the statistical analysis models. This study assessed the relationship between the availability of SAMU care in each municipality and the following 3 outcomes: the mortality due to AMI in the general population, AMI in-hospital mortality, and number of hospitalizations due to AMI, using the Poisson hierarchical model, and the analyzed rates were corrected by the age structure and detrended by temporal and seasonal influences. A 95% confidence interval (CI) was used for all outcomes.

## Results

AMI mortality rates, adjusted by age distribution, showed a decreasing trend throughout the study, ranging from 35.7 deaths per 100,000 inhabitants in 2008 to 30.4 deaths per 100,000 inhabitants in 2016, i.e., about 2% per year on average ( Table 1 ). Seasonal variation in AMI mortality rates was also observed, and it was higher during the winter season and lower during the summer season ( Figure 2 ).

**Table 1:** Annual AMI mortality, hospitalization, and in-hospital mortality rates, adjusted by age, in the state of Minas Gerais from 2008 to 2016

Year	Mortality (per 100,000)	In-hospital mortality (%)*	Hospitalization (per 100,000)
2008	35.7 (35.3 – 36.1)	13.81	152 (146 - 158)
2009	34.1 (33.8 – 34.5)	13.65	150 (144 - 156)
2010	35.0 (34.6 – 35.3)	13.78	140 (134 - 145)
2011	33.8 (33.4 – 34.1)	11.82	147 (142 - 152)
2012	32.4 (32.1 – 32.6)	11.29	146 (141 - 151)
2013	31.9 (31.7 – 32.2)	11.99	142 (137 - 146)
2014	30.9 (30.6 – 31.1)	12.15	137 (132 - 141)
2015	29.9 (29.7 – 30.2)	10.82	138 (133 - 142)
2016	30.4 (30.1 – 30.6)	11.43	147 (142 - 151)

*Rates are expressed with 95% confidence

**Figure 2:** Occurrence of seasonal variation in mortality rates from 2008 to 2016. A higher mortality rate is observed during winter, and lower rates are observed during summer.

Age-corrected AMI in-hospital mortality also showed a decreasing trend, from 13.81% in 2008 to 11.43% in 2016 ( Table 1 ), with wide monthly variations and a seasonal pattern, although this was less evident when compared to AMI mortality rate ( Figure 3 ).

**Figure 3:** Lethality and mortality monthly rates for the municipalities of Minas Gerais, from 2008 to 2016. Green dots represent monthly lethality rates for the whole analyzed population, which presents considerable monthly variation with no defined seasonal pattern. The red line represents the rate trend through time, with an accentuated reduction from 2008 to 2012, stagnation at 2013 and a new slower decrease from 2014 to 2016. We observed that the implementation of Mobile Emergency Care Service (SAMU), represented by the vertical blue lines, did not have any clear influence on lethality on the descriptive analysis.

SAMU implementation was associated with decreased AMI mortality (odds ratio [OR] = 0.967, 95% CI 0.936 to 0.998) and AMI in-hospital mortality (OR = 0.914, 95% CI 0.845 to 0.986) with no significant association with the number of hospitalizations (OR 1.003, 95% CI 0.927 to 1.083). There was no seasonal variation in the number of AMI hospitalizations during the study period.

## Discussion

In the present study, it was observed that the implementation of SAMU in the Brazilian state of MG was associated with a reduction in general AMI mortality rates. Age-corrected AMI in-hospital mortality also showed a decreasing trend over time, with a seasonal pattern. However, no significant association was found with AMI hospitalization rates during the analyzed period.

Despite the lack of evidence in developing countries, these results are in line with studies developed in high-income countries, and they are likely related to increased patient access to some type of AMI treatment and decreased time between symptom onset and initiation of specific therapy, including reperfusion when indicated.^3 , 8 , 17 , 18^ On the other hand, some studies have failed to demonstrate an association between the implementation of AMI systems of care and decreased mortality, despite the improvement in quality of healthcare, adherence to guideline-driven recommendations, and shortened treatment times.^3 - 5 , 8 , 12 , 19 , 20^ This has occurred even with the use of protocols designed with multiple interventions other than prehospital care only. Among these studies, the Reperfusion of Acute myocardial infarction in North Carolina Emergency departments (RACE) stands out. It was conducted in the state of North Carolina, USA, leading to a reduction in consultation time and the delays for reperfusion treatment administration following the implementation and systematization of protocols for AMI patient care.^20^

Differently from previous reports, our study was conducted in a middle-income country, a fact that can help to explain our positive results in terms of hard endpoints. In studies conducted in developed countries, the baseline quality of AMI care is generally superior to that observed in our country.^6 , 8 , 20^ Thus, demonstrating the magnitude of the incremental benefit of any intervention is more difficult to obtain in this situation, while in places where there is poor quality and care delivery, less complex interventions may have a greater impact on outcomes. A previous evaluation of adherence to AMI quality of care criteria in MG illustrates this care gap in the region.^21 - 23^ Despite the fact that the implementation of prehospital care was not part of the implementation of AMI systems of care, interventional facilities or dedicated cardiac care units in those regions, it may have influenced the improvement of the quality of care during the study period, since a higher proportion of patients might have received accurate diagnosis and appropriate treatment.

Outcomes associated with AMI are related to multiple variables, especially the structure for the care of patients.^22 - 24^ It is worth highlighting some relevant elements in the formulation of a high complexity network in cardiology: 1) the inverse relationship between the size and volume of procedures performed in referral hospitals and AMI mortality: 2) benefit of the “pharmaco-invasive” strategy, which consists of prehospital or early in-hospital fibrinolysis followed by routine PCI between 3 and 24 hours, comparable to primary PCI in patients with short-term AMI-related symptoms, whenever a timely transfer to a PCI-capable units is possible;^19^ 3) the cost-effectiveness of prehospital care expansion in relation to the construction of new interventional cardiology services, which was demonstrated in the USA, where the distance to hemodynamic services is around 70 km in the vast majority of sites.^1 , 2 , 20^

As expected, there was greater variation of rates in places with smaller populations, such as in the eastern and central-southern regions of the state, which have the lower HDIs of the state, respectively covered by the CISDESTE and CISRU consortia. This finding is possibly associated with the lower quality of the death certificate filling, with a higher proportion of deaths from undefined causes and “garbage codes” (health conditions that cannot be directly attributable to mortality), associated with the influence of other causes of mortality in those populations, such as infectious diseases, consistent with that observed in other low HDI regions in Brazil and worldwide (WHO, 2017).^24 - 26^ This finding reiterates the importance of using analytical adjustment methods for extreme variations. In this sense, the methodological analysis applying the global empirical Bayes method was effective in reducing the variation of rates in small populations, without changing the values in places with larger populations, as shown in Figure 4 . The use of the rates observed in the state of MG as a reference for smoothing allowed this process to be done with a reference population similar to the analyzed municipalities, but with a larger population.

**Figure 4:** Annual crude mortality rates in the state of Minas Gerais from 2008 to 2016. A temporal reduction trend.

With regards to seasonal variation in AMI mortality rates, this variation was not expected in a region with much lower temperature variation compared to North American or European countries, or even cooler regions of Brazil, such as São Paulo and the South Region. It raises the possibility of explanations other than the temperature, or temperature-related physiological changes, such as changes in blood viscosity, platelet volume, and blood pressure.^27^ Other diseases that are more common during the winter, especially respiratory infections by viruses and community-acquired pneumonia have shown to affect the cardiovascular system in various ways and to precipitate adverse cardiac outcomes, such as heart failure, myocardial infarction, and cardiac arrhythmias.^28^ As coronary artery disease is essentially an inflammatory disease, it has been shown that inflammation related to respiratory pathogens can trigger it.^29^ In a self-controlled case series using national infection surveillance data linked to the Scottish Morbidity Record, AMI and stroke rates substantially increased in the week following a respiratory infection.^30^ In addition, a recent meta-analysis has shown the impact of the 23-valent polysaccharide pneumococcal vaccine in providing protection from any cardiovascular event (risk ratio [RR]: 0.91; 95% CI: 0.84 to 0.99), myocardial infarction (RR: 0.88; 95% CI: 0.79 to 0.98), and all-cause mortality (RR: 0.78; 95% CI: 0.68 to 0.88) in individuals of all age groups.^31^ On the other hand, an ongoing clinical trial conducted in Brazil comparing single- to double-dose influenza vaccine after an acute coronary event was interrupted early due to apparent lack of benefit in interim analyses.^32^

Air pollution, especially exposure to particulate matter, has also been associated with a higher risk of cardiovascular diseases, including AMI.^33 , 34^ A recent study has observed that an increase of around 10 micrograms per m^3^of air was associated with an increase of 16% of AMI mortality.^33^ Meanwhile, another study using a global atmospheric chemistry model has shown that over 60% of cardiovascular deaths worldwide have been related to air pollution, and short-term increases in air pollution are associated to AMI.^34^ Sunyer et al., observed that the increase in sulfur dioxide levels in the air of seven European cities increased hospital admissions for cardiovascular diseases on the previous day and on the day of higher pollutant levels.^35^ This association remained significant even after adjusting for particles with size lower than 10 μm among subjects younger than 65 years. Air pollution tends to be higher in months in which rain levels are lower. Therefore, in Brazil, where the summers are rainy while winters have predominantly drier weather, seasonality may play an important role in AMI mortality indeed.

Our study has some limitations that should be addressed. The implementation of prehospital care is accompanied by co-interventions, such as articulation of the linkage of healthcare institutions of the AMI care system and increased local emergency services, which were not systematically studied in this study, and may have influenced the findings. Through public information available from the Ministry of Health, Minas Gerais State Health Secretariat, Municipal Health Secretariats and SAMUs’ databases, it is possible to affirm that the available networks were incipient and that there was a lack of infrastructure, supplies, human resources, and organizational and management processes in the majority of the state and the country.^11 , 13 , 14^ Examples include the low availability of interventional cardiology services and the concentration of health resources in regions with better sociodemographic profiles; the low utilization of telehealth services by prehospital teams; the absence of fibrinolytics in SAMU ambulances; the lack of clinical protocols and standardization of evidence-based care; the poor infrastructure of most emergency departments in the state, mostly prehospital emergency units and small hospitals; and the suboptimal training of health personnel involved in emergency care. Finally, these factors add to the difficulty of implementing the health care networks due to financial and political reasons.

This study did not address the impact of other levels of attention on the outcomes studied, such as primary health care (PHC). Although there are consistent data in the literature showing the key role of PHC in the prevention, promotion, and treatment of health conditions that are risk factors for AMI, with its ability to reduce the incidence rates of AMI, the focus of this study was acute events, which occur, albeit to a lesser extent, in well-established PHC systems.^1 , 2 , 6 , 8 , 20^ As the health effects associated with PHC are typically observed over the long term, no adjustment was made for this variable, given the technical difficulties inherent in this process and the scarcity of databases to perform it.

Another limitation is related to the fact that the ecological model does not include relevant individual clinical variables; therefore, it is not possible to establish relationships between such characteristics and the outcomes studied or to establish a definitive causal relationship between the implementation of SAMU and these outcomes. Finally, like all observational studies, the risk of bias has been minimized but cannot be completely excluded, in particular residual confounding bias.

Nevertheless, the major strength of our study is its methodological contribution, i.e., presenting a method that can take into account the seasonality and temporal trends to observe the effect of an intervention. The temporal trend of mortality reduction was taken into account in the analysis, and mortality reduction with SAMU implementation was observed regardless of this temporal trend.

## Conclusion

In the present study, a small reduction in general and in-hospital mortality rates attributable to AMI was observed following the implementation of SAMU in the Brazilian state of MG in the time frame analyzed, with no significant changes in hospital admission rates. Results suggest that prehospital care plays an important role in the healthcare system, especially considering the growing burden of cardiovascular diseases, especially acute coronary syndromes.

## * Supplemental Materials

For additional information, please click here.
